# Nondestructive, longitudinal, 3D oxygen imaging of cells in a multi-well plate using pulse electron paramagnetic resonance imaging

**DOI:** 10.1038/s44303-024-00013-7

**Published:** 2024-04-01

**Authors:** Safa Hameed, Navin Viswakarma, Greta Babakhanova, Carl G. Simon, Boris Epel, Mrignayani Kotecha

**Affiliations:** 1 Oxygen Measurement Core, O2M Technologies, LLC, 2201 W Campbell Park Dr, Chicago, IL 60612 USA; 2 Biosystems and Biomaterials Division, National Institute of Standards and Technology, 100 Bureau Drive, Gaithersburg, MD 20899 USA; 3 Department of Radiation and Cellular Oncology, The University of Chicago, 5841 S Maryland Ave, Chicago, IL 60637 USA

**Keywords:** Biotechnology, Biomaterials, Cellular imaging, Magnetic resonance imaging

## Abstract

The use of oxygen by cells is an essential aspect of cell metabolism and a reliable indicator of viable and functional cells. Here, we report partial pressure oxygen (pO_2_) mapping of live cells as a reliable indicator of viable and metabolically active cells. For pO_2_ imaging, we utilized trityl OX071-based pulse electron paramagnetic resonance oxygen imaging (EPROI), in combination with a 25 mT EPROI instrument, JIVA-25™, that provides 3D oxygen maps with high spatial, temporal, and pO_2_ resolution. To perform oxygen imaging in an environment-controlled apparatus, we developed a novel multi-well-plate incubator-resonator (MWIR) system that could accommodate 3 strips from a 96-well strip-well plate and image the middle 12 wells noninvasively and simultaneously. The MWIR system was able to keep a controlled environment (temperature at 37 °C, relative humidity between 70%–100%, and a controlled gas flow) during oxygen imaging and could keep cells alive for up to 24 h of measurement, providing a rare previously unseen longitudinal perspective of 3D cell metabolic activities. The robustness of MWIR was tested using an adherent cell line (HEK-293 cells), a nonadherent cell line (Jurkat cells), a cell-biomaterial construct (Jurkat cells seeded in a hydrogel), and a negative control (dead HEK-293 cells). For the first time, we demonstrated that oxygen concentration in a multi-well plate seeded with live cells reduces exponentially with the increase in cell seeding density, even if the cells are exposed to incubator-like gas conditions. For the first time, we demonstrate that 3D, longitudinal oxygen imaging can be used to assess cells seeded in a hydrogel. These results demonstrate that MWIR-based EPROI is a versatile and robust method that can be utilized to observe the cell metabolic activity nondestructively, longitudinally, and in 3D. This approach may be useful for characterizing cell therapies, tissue-engineered medical products, and other advanced therapeutics.

## Introduction

Cell and gene therapy, tissue engineering, and regenerative medicine hold promise to make breakthroughs in many medical fields, such as cancer, type I diabetes, sickle cell disease, musculoskeletal damage, trauma, and wound healing^1–4^. These fields rely on autologous or allogeneic functional and viable cells for repair and regeneration^5–7^. Although promising, many hurdles remain to utilize the full potential of these advanced therapies^8^. Hypoxia is a major bottleneck. It has been shown that adequate oxygenation is the key to the success of beta cell replacement therapies^9–15^, CAR-T cell therapies^16–18^, musculoskeletal regeneration^19–22^, and almost all tissue regeneration and growth. Multiple approaches are being employed across the fields to overcome hypoxia and to keep cells viable and functional for therapy purposes before, during, and after implantation. Another challenge is the assessment of cell viability without destroying the product. Currently, no method can provide a quantitative measure of viable cells without destroying them in the process. The issue is exacerbated if the cells are encapsulated in a scaffold. Ideally, the metabolic activities of cells should be assessed via a non-destructive, 3D, and longitudinal method before and after implantation.

The use of oxygen by cells and tissues is a fundamental aspect of basic redox biology. Oxygen consumption is the major component of cellular respiration and is a direct indication of viable and functional cells^9^. The amount of oxygen utilized by cells varies across species, cell types, and metabolic pathways^9,23^. Therefore, oxygen measurement in situ can provide key information on cell health. Fluorescence-based oximetry can provide single-point oxygen measurements for cell-containing constructs. However, oxygen imaging has the advantage of providing spatiotemporal information regarding local oxygen concentrations^24^. Oxygen imaging methods, such as blood-oxygen-level dependent magnetic resonance imaging (BOLD MRI) and photoacoustic imaging, rely on the vascular structure or paramagnetic properties of hemoglobin making them unsuitable for *in vitro* oxygen measurements.

**Fig. 1:**
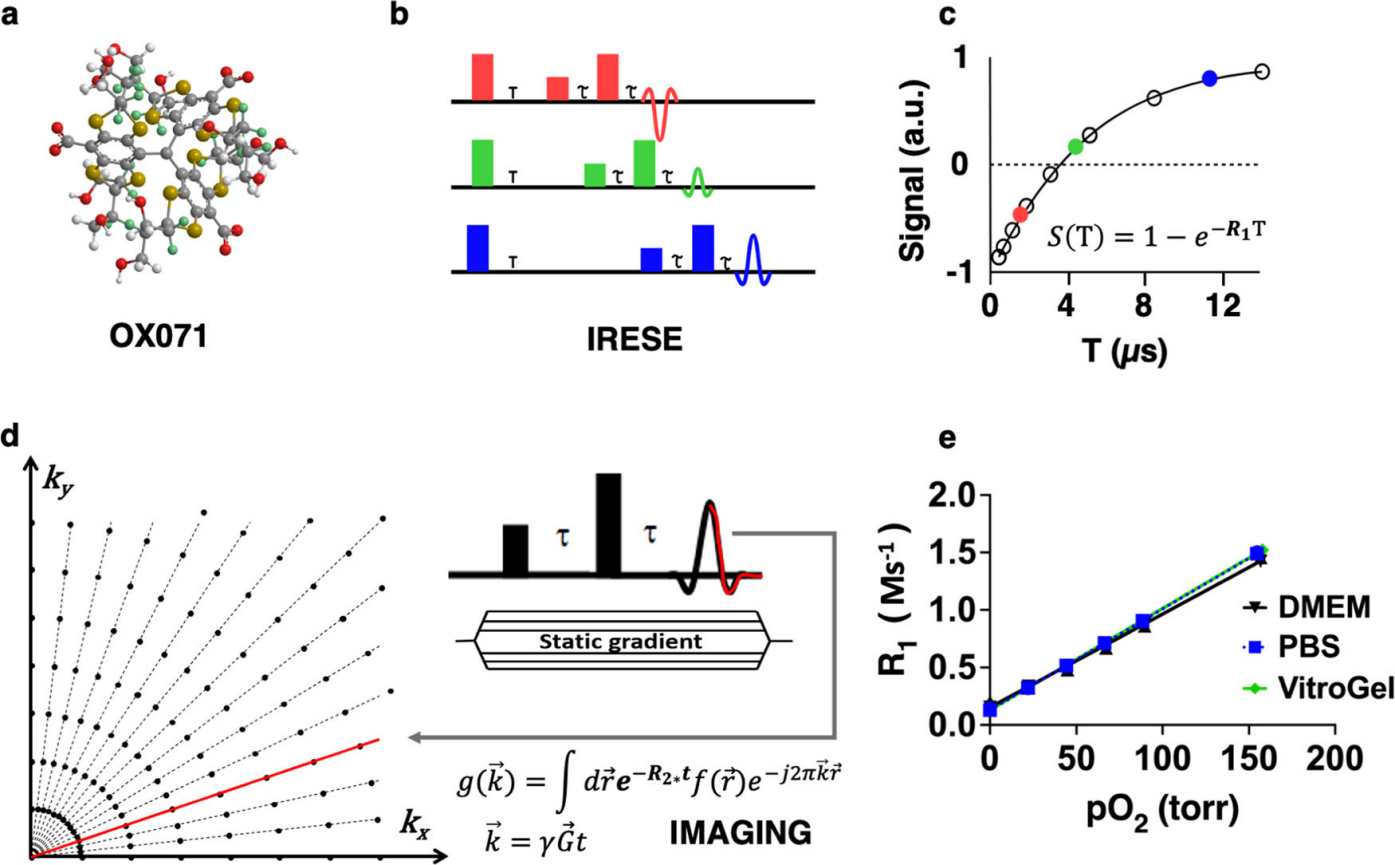
Schematic of EPROI. **a** Ball-and-stick model of oxygen sensitive spin probe, OX071. Carbon atoms are depicted in grey, hydrogen in white, deuterium in green, oxygen in red, and sulfur in gold. **b** Inversion recovery electron spin echo pulse sequence. **c** single exponential recovery fit of the signal as a function of delay, T, that is used to calculate R_1_ (= 1/T_1_). **d** radial *k*-space acquisition scheme shown in 2D with static gradients that are used for image acquisition. **e** R_1_ vs pO_2_ calibration curves for the materials used in the current project. The slopes and intercepts are: 109.664 torr/Ms^−1^, 0.181 Ms^−1^; 123.3941 torr/Ms^−1^, 0.1537 Ms^−1^; 113.484 torr/Ms^−1^, 0.130 Ms^−1^ for PBS, DMEM cell culture medium, and VitroGel, respectively.

Pulse electron paramagnetic resonance (EPR) oxygen imaging (EPROI) is an emerging oxygen imaging method that provides 3D partial pressure of oxygen (pO_2_) with high spatial, temporal, and pO_2_ resolution^24–30^. EPROI, where unpaired electrons are probed, is similar to MRI (can be called eMRI or electron MRI), but with a few key differences: (a) it uses a lower magnetic field (typically 9 to 42 mT) due to the 658 times higher magnetic moment of electrons compared to protons, and (b) it requires exogenous contrast agents due to the extremely short lifetimes and relaxation rates of EPR-sensitive endogenous probes in the body. For the past two decades, the trityl radical OX071 (Fig. 1a, chemical formula: C_52_H_39_D_24_O_18_S_12,_ molecular weight: 1384.97) has been used for EPROI because of its useful EPR properties (stable with narrow line width and long relaxation time, in the range of 1–10 μs) and suitable biophysical properties (high-water solubility, non-toxic nature)^24,31^. The spin-lattice relaxation rate (R_1_ = 1/T_1_) of trityl OX071 and its linear relationship with pO_2_ are utilized for oxygen imaging^27^. The R_1_ is measured using the inversion recovery electron spin echo (IRESE) (Fig. 1b) pulse sequence with a single exponential recovery fitting (Fig. 1c) and imaged using a 3D radial acquisition scheme and static gradients (Fig. 1d). Filtered back projection (FBP) reconstruction is utilized for image reconstruction^31–36^. Trityl, which does not consume oxygen during oxygen reporting or enter cells, is an ideal molecule to report extracellular pO_2_^37^. EPROI has high sensitivity (<1 torr) under hypoxic conditions and lower sensitivity (~3–5 torr) at 21% oxygen^24^. The reduced sensitivity at high oxygen conditions is due to reduced signal-to-noise ratio (SNR) caused by increased line broadening of the trityl EPR line, leading to increased error in the fit for the T_1_ calculation^27^. Fig. 1e provides the relationship between R_1_ and pO_2_ for materials used in the current study (PBS, culture medium, and VitroGel). For the past two decades, EPROI has been primarily used for cancer hypoxia research with few notable results, such as the demonstration of oxygen-guided radiation therapy in three different tumor models and rabbit VX-2 tumor pO_2_ imaging using a human-sized EPROI instrument^38–43^. Recently, the applications of EPROI have been extended to the assessment of biomaterials, engineered grafts, and cell encapsulation devices^13,14,24,30,44–48^.

In this work, our goal was to develop an incubator-resonator for a multi-well plate and perform oxygen imaging of live cells while using a custom-built incubator to provide physiological conditions (temperature, humidity, CO_2_) during imaging. Although EPROI is not restricted to any specific size or shape of an object, the multi-well plate was chosen because it is a standard consumable in biological labs and allows multiple replicates to be assessed simultaneously to increase statistical power. We used our recently introduced 25 mT instrument, JIVA-25™, for oxygen imaging. The instrument has a 10 cm bore gap and is suitable for oxygen imaging *in vitro* and in rodents^13,14,24,49,50^. Most EPROI resonators for in vitro and in vivo oxygen imaging are cylindrical. The largest EPROI resonators to date are 32 mm in diameter for mice (JIVA-25^TM^) and 57 mm for a rabbit leg (for a 9 mT human-size EPROI instrument)^43,44,51^. Larger resonators are a challenge because the signal-to-noise ratio (SNR) drops as a square root of volume (SNR ∝ 1/sqrt(V))^24,43,44^. To investigate the oxygen concentration of cells in a multi-well plate in a controlled environment, we designed an environment-controlled multi-well plate incubator resonator (MWIR) that was rectangular for the best filling factor.

The ability of MWIR to perform oxygen imaging of live cells was tested using an adherent cell line (HEK-293), a nonadherent cell line (Jurkat cells), and Jurkat cells seeded in a hydrogel. For the experiments with cells, two identical sets of wells were prepared by seeding cells into strip wells. One set was used for oxygen imaging in the MWIR, while the second set was placed inside the standard biological-oxygen demand (BOD) incubator as a control. We performed oxygen imaging of cells with varying densities to assess the sensitivity of the method. We performed longitudinal oxygen imaging to assess the change in cell metabolism as a function of time. We also assessed dead cells as a negative control. We demonstrate oxygen imaging of live cells as a method to non-invasively assess their viability in 2D and 3D culture.

## Results

**Fig. 2:**
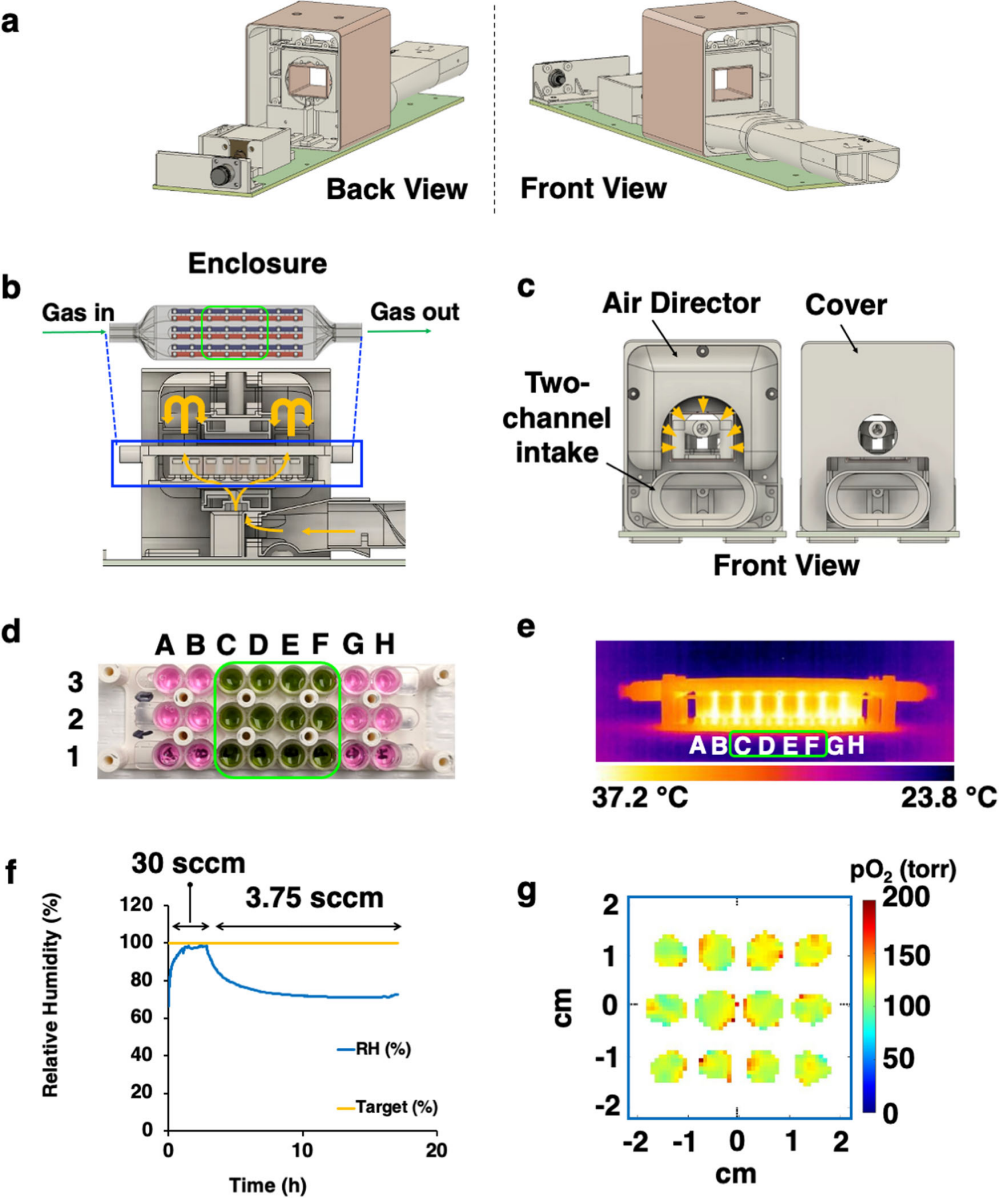
Schematics of the MWIR apparatus. **a** A rectangular resonator without air directors and covers. **b** a cross-section of multi-well plate enclosure and the resonator, air flow is shown using yellow direction arrows. **c** a resonator with air director (left) and cover (right) installed; a two-channel air intake is visible. **d** Three 8-well stripwells from a 96-well plate with columns C to F containing 150 μL of culture medium with 1 mM OX071. The 12 wells outlined in green were used for pO_2_ imaging. **e** Thermal image of the multi-well plate enclosure immediately after removal from MWIR showing that the temperature was maintained at 37 °C. **f** Relative humidity plot shows that a gas flow rate of 30 sccm sets the humidity at 100% while 3.75 sccm sets the humidity at 70%. **g** Representative pO_2_ maps (slice #4 at ~ 3.2 mm from the bottom of the well) of the middle 12 wells (columns C to F) filled with PBS during reoxygenation with 95% air + 5% CO_2_ after N_2_ bubbling. SCCM (standard cubic centimeter per min).

Our first goal was to design hardware for environmental control during oxygen imaging of cells in 96-well strip-well plates by controlling temperature, CO_2_, and humidity. Recently, we introduced a 25 mT (720 MHz resonance frequency) EPROI instrument JIVA-25™, and we designed and tested a multi-well plate incubator resonator (MWIR) for the instrument. Figure 2 shows the schematics and components of MWIR. JIVA-25™ has a 10 cm air gap between the magnet poles where the MWIR was to be placed. The MWIR has three components: (a) a rectangular resonator for oxygen imaging (Fig. 2a) (b) a multi-well plate enclosure to keep the cells at desired high humidity and gas (95% air + 5% CO_2_) conditions (Fig. 2b, c), and (c) heating control to maintain the temperature of 37 °C (not shown).

Resonators are special volumetric units in magnetic resonance instruments that confine radiofrequency energy and allow it to interact with the studied object^52–54^. Typical EPROI resonators are cylindrical. However, to accommodate a multi-well plate, we designed and tested a rectangular 2-gap loop-gap resonator of 42 mm L × 38 mm W × 26 mm H (Fig. 2a). This is one of the largest volume resonators used in the field of EPROI. The cross-section of this resonator can accommodate three strip wells of a 96-well strip-well plate (Fig. 1D). Only the middle 12 wells (3 × 4) fit inside the volume of the resonator are imaged. The rest of the wells, while being maintained at the constant temperature, humidity, and gas conditions, are outside the imaging volume.

The second component (Fig. 2b, c) is the sealed multi-well plate enclosure, with precisely controlled delivery of the necessary gas mixtures. For all experiments, the environment was maintained at a humidified state with 95% air plus 5% CO_2_. The system can accommodate any other gas conditions, such as 5% O_2_. An integrated two-channel (hot-cold) control system was implemented to maintain the temperature at 37 °C. Ducts and air directors were built into the MWIR to enable air circulation from all sides of the plate enclosure (Fig. 2b, c).

The MWIR can image the 12 middle wells of 3 single strips from a 96-well stripwell plate (Fig. 2d), while keeping the temperature constant at 37 °C ± 1 °C (Fig. 2e) and relative humidity (RH) between 70% and 100% (Fig. 2f) by controlling the flow rate of the gas mixture between 3.75 sccm (standard cubic centimeter per min) to 30 sccm. A high gas flow rate maintained high humidity, but the system became susceptible to condensation in some of the wells, which affected cell viability beyond 8 h. Reducing the gas flow rate eliminated condensation without compromising cell viability. Therefore, a gas flow rate of 3.75 sccm was used for all experiments with cells. The MWIR was tested for its ability to provide a controlled environment for oxygen imaging using PBS in the middle 12 stripwells (Fig. 2g).

**Fig. 3:**
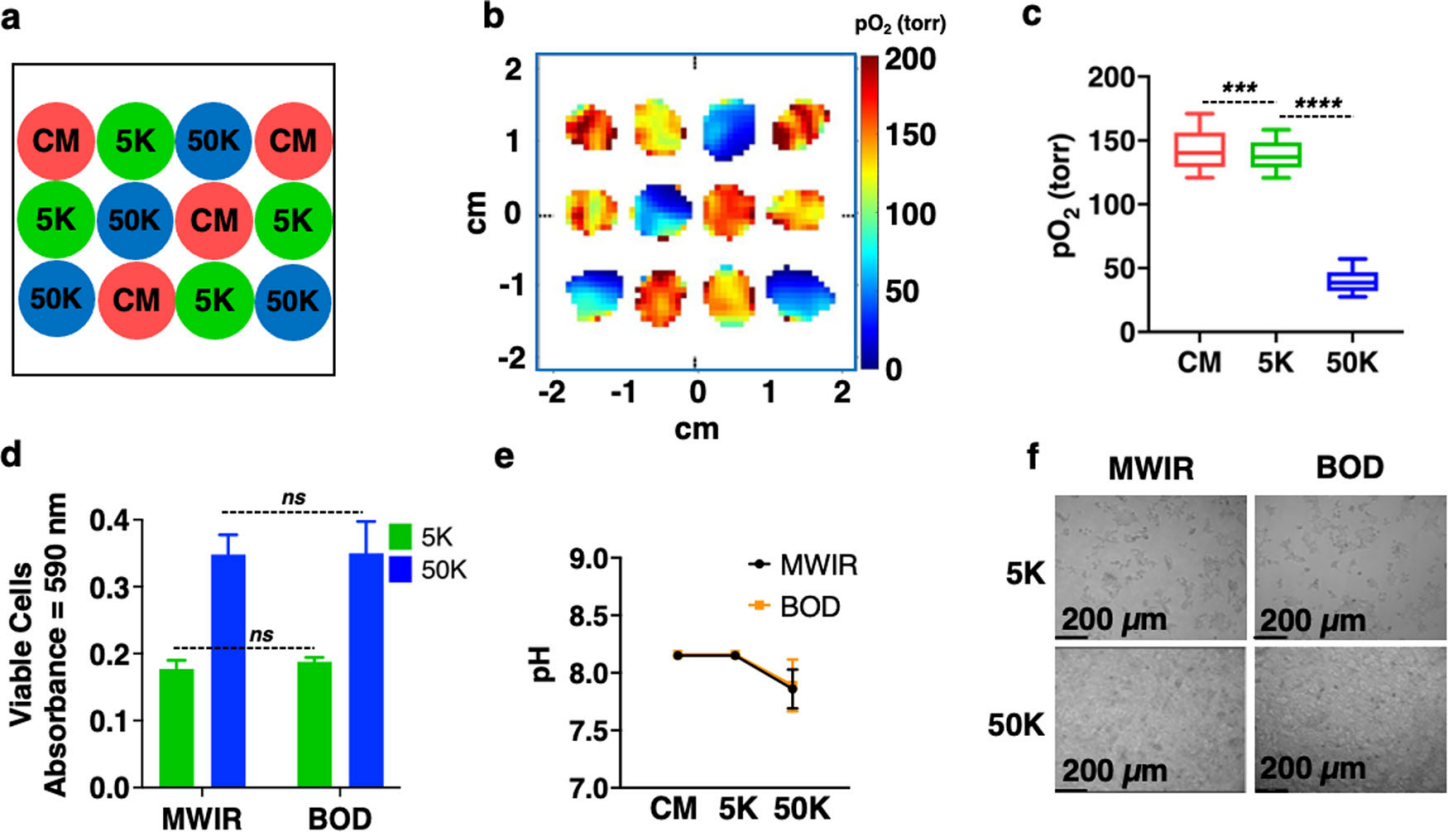
Oxygen imaging of HEK-293 cells in MWIR. **a** The pattern of cell seeding for control (CM), 5 K, and 50 K cells per well (*n* = 4). Each well had 150 μL of medium. **b** Representative pO_2_ maps of wells (slice #4, ~3.2 mm from the bottom of the well) in the transverse plane at t = 0 h (first imaging experiment). **c** Box plot of pO_2_ for each cell density; differences are statistically significant (*p* ≤ 0.001). **d** Cell viability measured by MTT assay after 24 h of imaging shows no significant difference between cells in the MWIR and BOD incubator at 5 K and 50 K densities (*p* > 0.05). **e** pH of the medium from wells that were in MWIR and in the incubator after 24 h. **f** Cell morphology after 24 h imaging from MWIR and BOD.

For experiments with cells, two identical sets of wells were prepared by seeding HEK-293 cells into strip wells. One set was used for oxygen imaging in the MWIR, while the second set was placed inside the BOD incubator as a control. Three cell densities were used in quadruplicate: control medium with no cells (CM), 5000 cells per well (5 K), and 50,000 cells per well (50 K). The cells were arranged in the middle 12 wells as shown in Fig. 3a. Figure 3b shows the representative pO_2_ maps from these middle 12 wells at the beginning of the experiment. Interestingly, the wells with 50 K cells had lower oxygen tension than CM or 5 K cells, even though all wells were individually supplied with 19.95% O_2_ gas flow. The lower oxygen tension for wells with 50 K cells represents a higher oxygen demand. Figure 3c provides the box plots of pO_2_ values vs cell density at t = 0. The difference in pO_2_ values between CM and 5 K cells (*p*-value = 0.0002) and between 5 K cells and 50 K cells (*p*-value < 0.0001) is statistically significant, showing that pO_2_ maps can be used to assess cell viability.

We performed the oxygen imaging experiments for 2 h, 4 h, 8 h, and 24 h and compared cell viability for the MWIR versus BOD using the MTT assay (3-(4,5-dimethylthiazol-2-yl)-2,5-diphenyltetrazolium bromide) for cellular dehydrogenase activity. The pO_2_ images and cell viability using MTT assay for MWIR versus BOD are similar for all time points and no significant differences were observed (Fig. 3d). The pH values (Fig. 3e) and cell morphology (Fig. 3f) of cells in the MWIR system were also consistent with the BOD incubator. These results demonstrate that EPROI imaging in the MWIR does not affect cell viability or metabolic activity during a 24 h experiment.

**Fig. 4:**
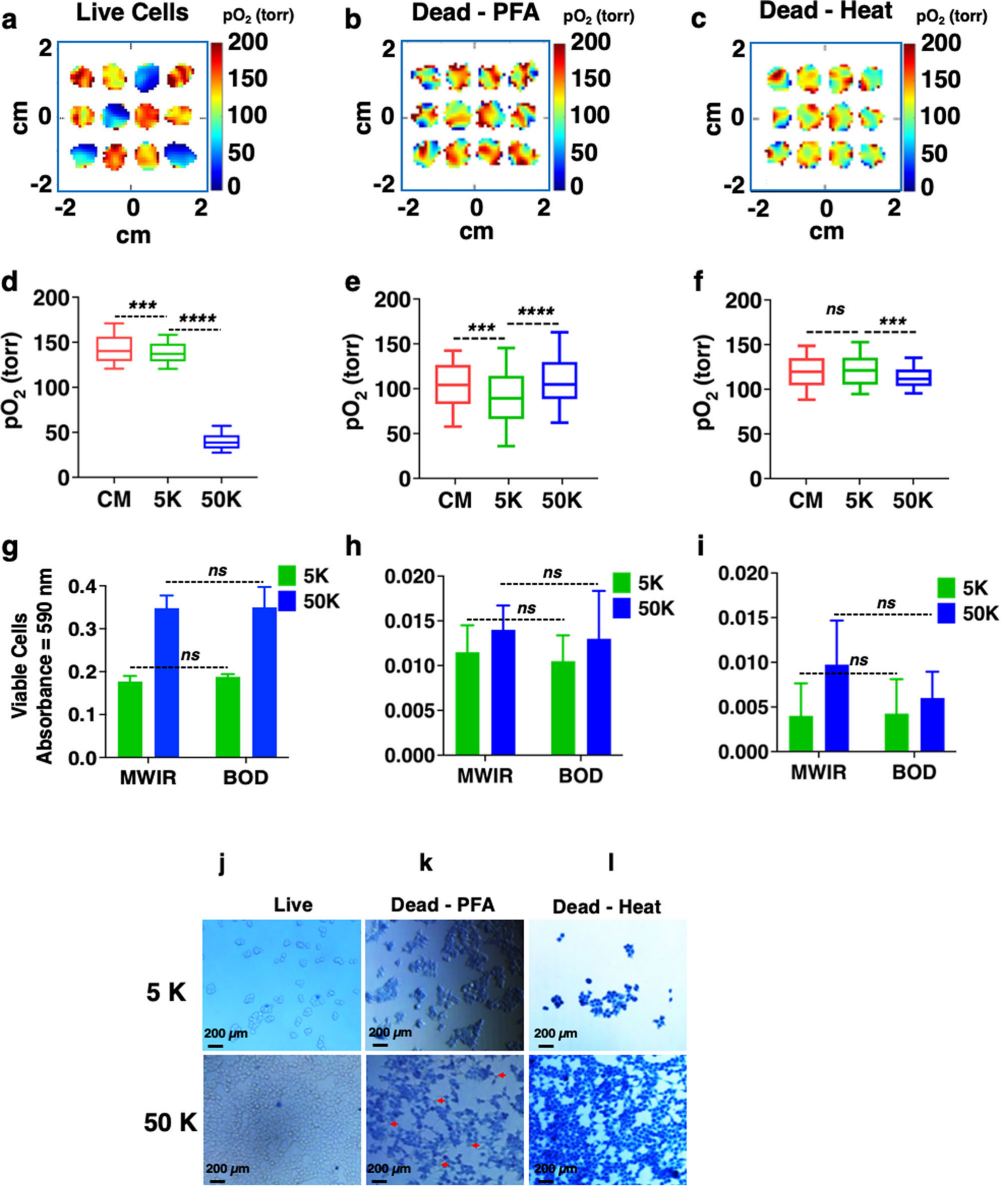
Comparison of pO_2_ of live and dead HEK-293 cells. The cells were seeded in the same pattern shown in Fig. 3a. Representative pO_2_ maps and box plots of pO_2_ values as a function of cell density (**a**, **d**) live cells (same as Fig. 3a), (**b**, **e**) dead cells (killed with 4% PFA), and (**c**, **f**) dead cells (killed with heat-shock). A significant difference in the pO_2_ of 5 K and 50 K cells per well is observed for all three cases. **d** (live cells) CM vs 5 K: *p* = 0.0002; 5 K vs 50 K: *p* < 0.0001. **e** (PFA dead cells) CM vs 5 K: *p* = 0.0008; 5 K vs 50 K: *p* < 0.0001. **f** (Heat-shock) CM vs 5 K: *p* = 0.2598; 5 K vs 50 K: *p* < 0.0001. Note that the medium was also treated with PFA or heat for the dead cell experiments. **g**–**i** MTT assay of live and dead cells after imaging shows no significant difference between MWIR and BOD (*p* > 0.05). **j**–**l** Trypan blue exclusion assay for the confirmation of cell death before imaging. Some cells were still alive when killed using 4% PFA (live cells are indicated by red arrows).

Next, control experiments using dead HEK-293 cells were performed to verify oxygen imaging results. The same three seeding densities and seeding patterns were used as in Fig. 3a. The cells were killed either by exposing them to 4% paraformaldehyde (PFA) for 30 min or to 70 °C for 15 min, as confirmed by morphological assessment. The pO_2_ images of dead cells were taken immediately after killing the cells. As expected, the difference in pO_2_ maps of dead cells with 0 (CM), 5000 (5 K), and 50,000 (50 K) cells per well is less pronounced but statistically significant compared to the live cells in both sets, as shown in Fig. 4a, c (live cells), b & d (dead with PFA) and c & g (dead with heat-shock). Note that the medium was also treated with heat or PFA for dead cell experiments. The MTT assay (Fig. 4g–i) shows no significant difference between the MWIR and BOD. Figure 4j–l shows trypan blue staining for assessing cell morphology and viability. Some cells are still alive after PFA treatment (Fig. 4k), which could explain the lower pO_2_ in the wells for this treatment. It is possible that dead cells and the treatment of the medium with PFA or heat-shock change the medium viscosity and oxygen permeability in this medium, which is reflected by the statistically significant difference in pO_2_ values for the dead cells. These results confirm that pO_2_ imaging is a sensitive tool that can differentiate between live and dead cells.

**Fig. 5:**
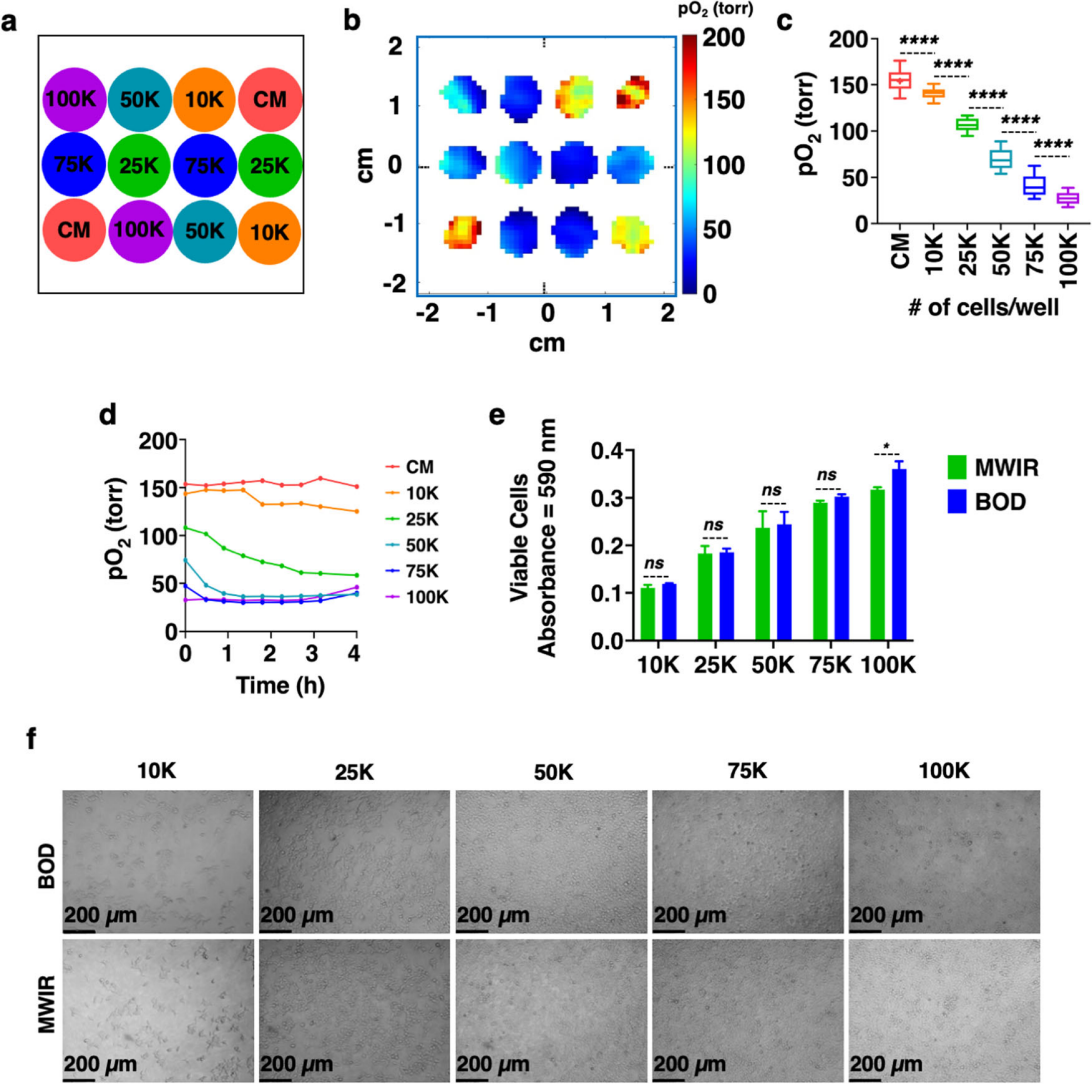
The sensitivity of pO_2_ imaging. **a** Six different cell densities of HEK-293 cells (CM (0 K), 10 K, 25 K, 50 K, 75 K, and 100 K cells per well) were seeded in the given pattern (*n* = 2). **b** A representative pO_2_ map at t = 0 h showing visual assessment of cell metabolic activity. The image is taken ~3.2 mm above the bottom of the well. **c** Box plot of pO_2_ as a function of cell density. The mean and standard error obtained from the pO_2_ map for CM (144.06 ± 4.82 torr), 10 K (116.61 ± 1.45 torr), 25 K (49.58 ± 0.74 torr), 50 K (27.97 ± 0.70 torr), 75 K (30.15 ± 0.94 torr), and 100 K (32.15 ± 1.18 torr), (*n* = ~ 200–300 voxels per well, *n* = 2 per cell density). There is a statistically significant difference (*p* < 0.0001) in pO_2_ between CM vs 10 K, 10 K vs 25 K, 25 K vs 50 K, 50 K vs 75 K, and 75 K vs 100 K. **d** The change in pO_2_ as a function of time. **e** MTT assay after 4 h revealed no significant differences between MWIR and BOD (*p* > 0.05), except for 100 K cells (*p* ≤ 0.05). **f** Morphological assessment of cells in MWIR and BOD after 4 h of measurements.

Next, the sensitivity of oxygen imaging to cell density was assessed. HEK-293 cells were seeded at six different densities: 0 (CM), 10,000 (10 K), 25,000 (25 K), 50,000 (50 K), 75,000 (75 K), and 100,000 (100 K) cells per well. Figure 5a shows the arrangement of cells in the wells with two replicates of each cell density. Figure 5b shows the representative pO_2_ maps of these wells and confirms that pO_2_ mapping can differentiate between these cell densities. The box plot at t = 0 shown in Fig. 5c shows that the wells with higher cell densities had lower pO_2,_ and the difference between different cell densities is statistically significant. Figure 5d shows how the average pO_2_ evolved over the course of 4 h. As cells differentiate, proliferate, become dormant, or die, their oxygen consumption rate changes, which can be reflected in pO_2_ values. Most interesting are the time trends of 50 K, 75 K, and 100 K cells per well. We can see that within 4 h, the mean pO_2_ values of wells with 50 K cells matched the pO_2_ of wells with 100 K cells, where cell dormancy or cell death was probably happening because of overcrowding. The pO_2_ for 25 K cells dropped over time_,_ indicating increased metabolic activity in these wells. A comparison of the MTT assay for cells in the MWIR with cells in the BOD incubator shows no significant difference in cell viability except for 100 K cells per well (Fig. 5e). These experiments demonstrate the ability of EPROI to observe longitudinal cellular oxygen consumption and to differentiate between six different cell densities. One limitation of pO_2_ imaging technology could be when cells change their metabolism due to the environment; they may show similar pO_2_ as is the case for 50 K, 75 K, and 100 K cells in Fig. 5d. A similar cell morphology was observed for cells at all seeding concentrations after 4 h in the MWIR or BOD (Fig. 5f).

**Fig. 6:**
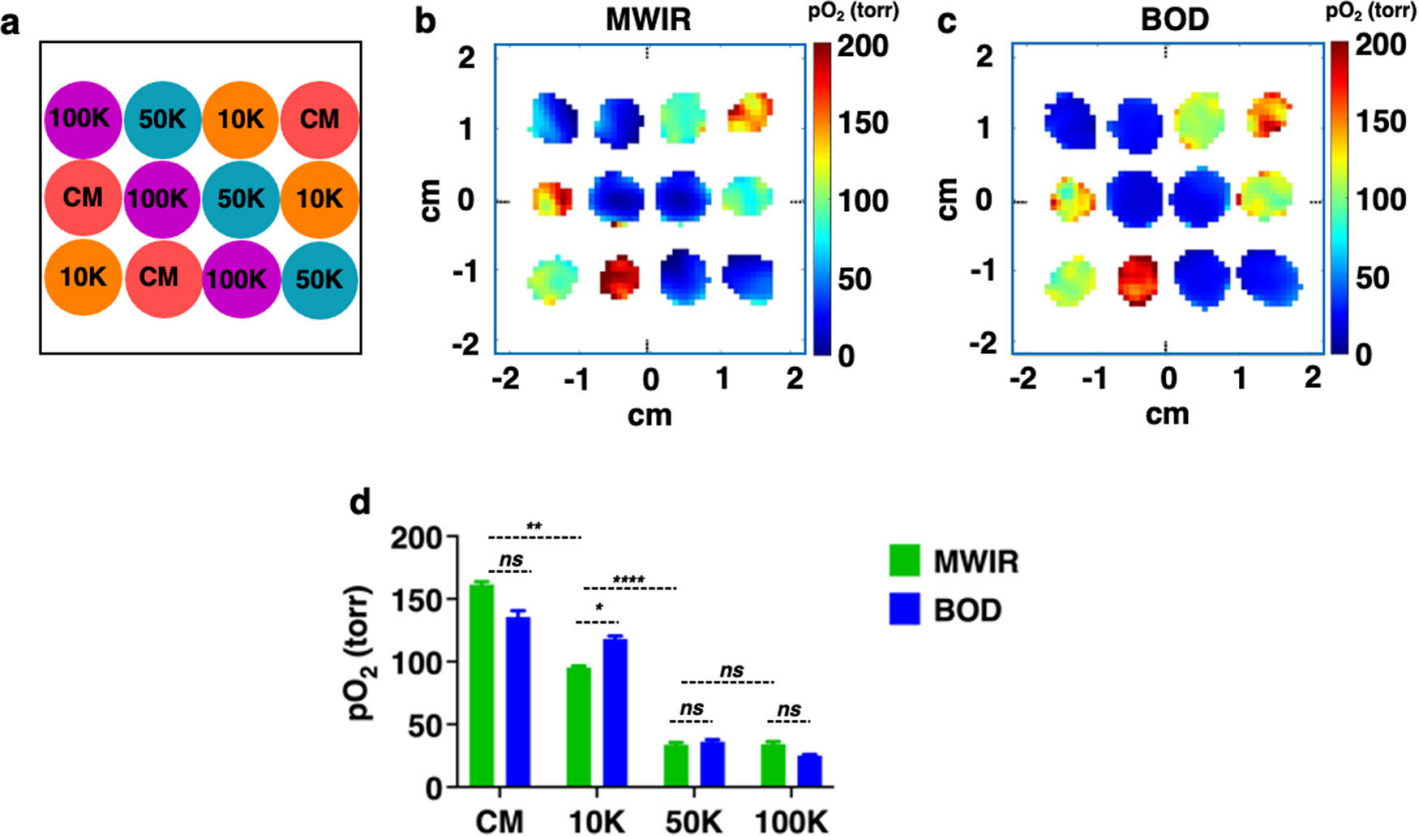
Assessment of pO_2_ for HEK- 293 cells cultured 4 h in the BOD incubator. **a** The layout of the wells for 0 K (CM), 10 K, 50 K, and 100 K cells per well (*n* = 3). Representative pO_2_ maps at 4 h of cells in (**b**) MWIR and (**c**) BOD incubator taken ~ 3.2 mm from the bottom of the wells. **d** pO_2_ quantification between the MWIR and BOD incubator. The mean and standard error of pO_2_ are: MWIR- CM (148.27 ± 2.97 torr), 10 K (91.77 ± 1.16 torr), 50 K (34.9 ± 0.95 torr), 100 K (35.98 ± 0.99 torr) or BOD -CM (106.42 ± 1.00 torr), 10 K (110.69 ± 1.85 torr), 50 K (37.10 ± 1.57 torr), 100 K (25.95 ± 1.05 torr), (*n* = 300–750 voxels per well, *n* = 3 per cell density). There is a significant difference (*p* < 0.0001) in pO_2_ between different cell densities. The MWIR vs BOD *p*-values among wells are as follows: CM, *p* = 0.1938; 10 K, *p* = 0.0179; 50 K, *p* = 0.5495; 100 K, *p* = 0.01863; MWIR: CM vs 10 K, *p* = 0.0015; 10 K vs 50 K, *p* = 0.00014; and 50 K vs 100 K *p* = 0.9415.

The experiments in Fig. 5 show that cell viability was similar for the MWIR and BOD, even though low pO_2_ was observed for high-cell density wells (50 K, 75 K, 100 K). This raised a question of whether the high cell density wells in the BOD incubator were also experiencing low pO_2_. This was tested by the following experiment. Two identical sets of plates with four cell densities (0 K (CM), 10,000 (10 K), 50,000 (50 K), and 100,000 (100 K) cells per well, triplicate for each cell density) were made. One was placed in the MWIR and the other in the BOD incubator and, after 4 h, pO_2_ images were acquired for both sets. Figure 6a shows the arrangement of cells in the middle 12 wells in the plate, and Fig. 6b, c shows the representative pO_2_ maps of the cells in MWIR and in the BOD incubator for 4 h, respectively. Interestingly, similar to MWIR, the cells in the BOD incubator also experienced low pO_2_ for high cell densities. Figure 6d shows the pO_2_ values for MWIR and BOD. In all cases, there are no significant differences between MWIR and BOD (except 10 K, *p ≤ 0.05).

**Fig. 7:**
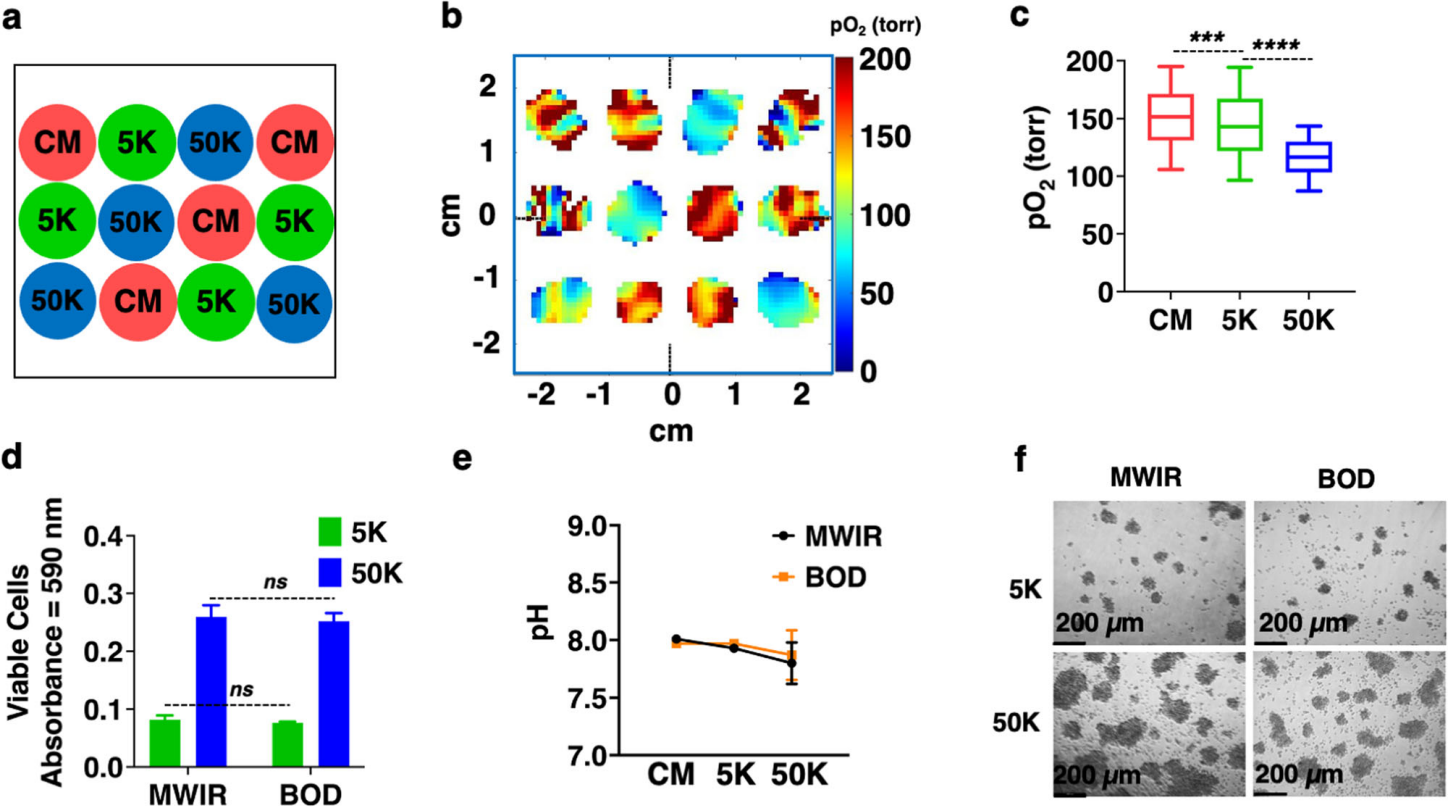
Oxygen imaging of Jurkat cells using MWIR. **a** The cell seeding pattern of Jurkat cells CM, 5 K, and 50 K cells per well (*n* = 4). **b** A representative pO_2_ map taken at t = 0 h (~ 3.2 mm from the bottom of the well). **c** The box plot of pO_2_ values from t = 0 h. Differences in pO_2_ are statistically significant: CM vs 5 K, *p* = 0.0002 and 5 K vs 50 K, *p* < 0.0001. **d** The MTT assay after 4 h of imaging showed no significant difference in cell viability between the MWIR and BOD incubator (*p* > 0.05). **e**, **f** pH and cell morphology confirm that the MWIR emulates incubator-like conditions to enable in situ oxygen mapping of live cells.

The above experiments were performed using HEK-293 adherent cells that were attached to the bottom of the well. Next, we performed oxygen imaging of a non-adherent cell line, Jurkat cells, using MWIR. Cell densities of 0 K (CM) 5 K, and 50 K cells per well were used in the arrangement shown in Fig. 7a. Representative pO_2_ maps (Fig. 7b) and box plots (Fig. 7c) at t = 0 show higher pO_2_ values for these cells compared to HEK-293 (Fig. 3b, c), indicating different metabolic activity for Jurkat cells. The pO_2_ heterogeneity may be due to the Jurkat cells growing in aggregates. The MTT viability assay (Fig. 7d), pH test (Fig. 7e), and cell morphology (Fig. 7f) after 4 h of oxygen imaging show that there is no significant difference between the Jurkat cells in MWIR versus the BOD incubator, and EPROI using the MWIR is a noninvasive and robust method.

**Fig. 8:**
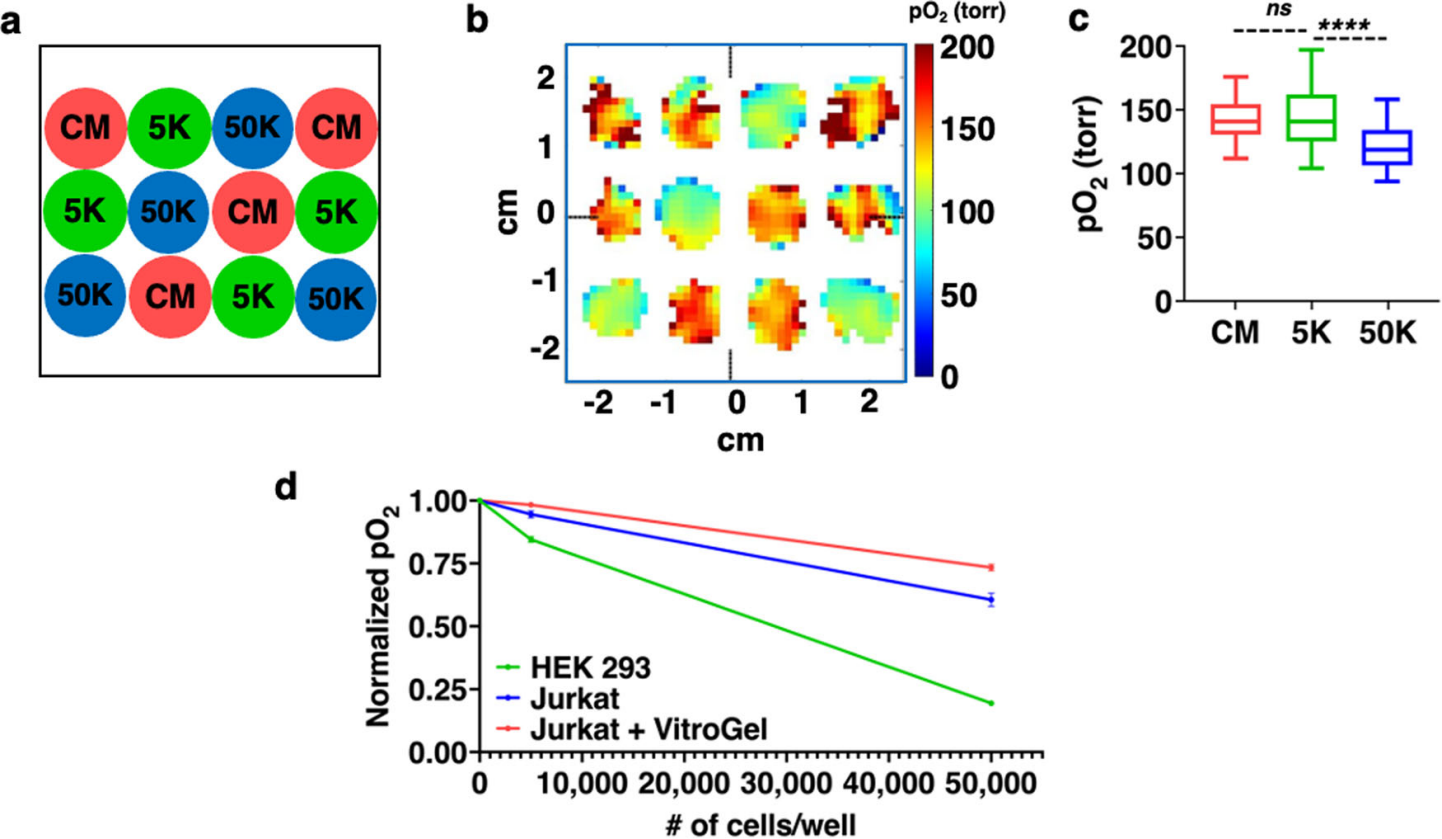
Oxygen imaging of Jurkat cells seeded in VitroGel. **a** The cell seeding pattern for 0 K (CM), 5 K, and 50 K cells per well (*n* = 4). **b** pO_2_ maps of the wells at time t = 0 (~3.2 mm from the bottom of the wells). **c** Box plot of pO_2_ vs cell density at t = 0. There is a statistically significant difference between 5 K and 50 K cells (*p* < 0.0001), but no significant difference between CM and 5 K cells (*p* = 0.6581). **d** Plot of pO_2_ versus cell density (0 K, 5 K, and 50 K cells per well, *n* = 4) for HEK-293, Jurkat, and Jurkat in VitroGel. Error bars are standard errors.

A significant issue for assessing tissue-engineered medical products is that once the cells are seeded into a hydrogel scaffold, there is no way to track their metabolic activity without destroying the construct. To address this issue, we performed oxygen imaging of Jurkat cells seeded in a VitroGel, a commercially available glucose-based hydrogel. The cell seeding scheme with three densities, 0 K (CM), 5 K, and 50 K cells per well is shown in Fig. 8a. Figure 8b shows the representative pO_2_ maps. The box plot in Fig. 8c shows that the pO_2_ values were significantly different for the wells with 5 K and 50 K cells. Interestingly, the pO_2_ values are higher for Jurkat cells in VitroGel (Fig. 8c) compared to Jurkat cells in culture medium (Fig. 3c), suggesting that Jurkat cells have reduced metabolic activity upon cell-seeding or that cells are lost during the VitroGel encapsulation process.

Figure 8d shows the normalized pO_2_ values for HEK-293, Jurkat, and Jurkat in VitroGel as a function of cell seeding density. The normalized pO_2_ values were calculated by dividing the mean pO_2_ of wells with cells by the mean pO_2_ of wells with control medium. The normalized pO_2_ of Jurkat cells in VitroGel is higher than Jurkat cells in medium or HEK-293 cells. HEK-293 cells have the lowest pO_2_ or the highest oxygen consumption rate (OCR). For Jurkat cells in VitroGel, the metabolic activity of cells is reduced by 17.3% for 50 K cells and by 4% for 5 K cells compared to Jurkat cells in medium, providing a quantitative assessment of the impact of cell seeding on viable cells.

**Fig. 9:**
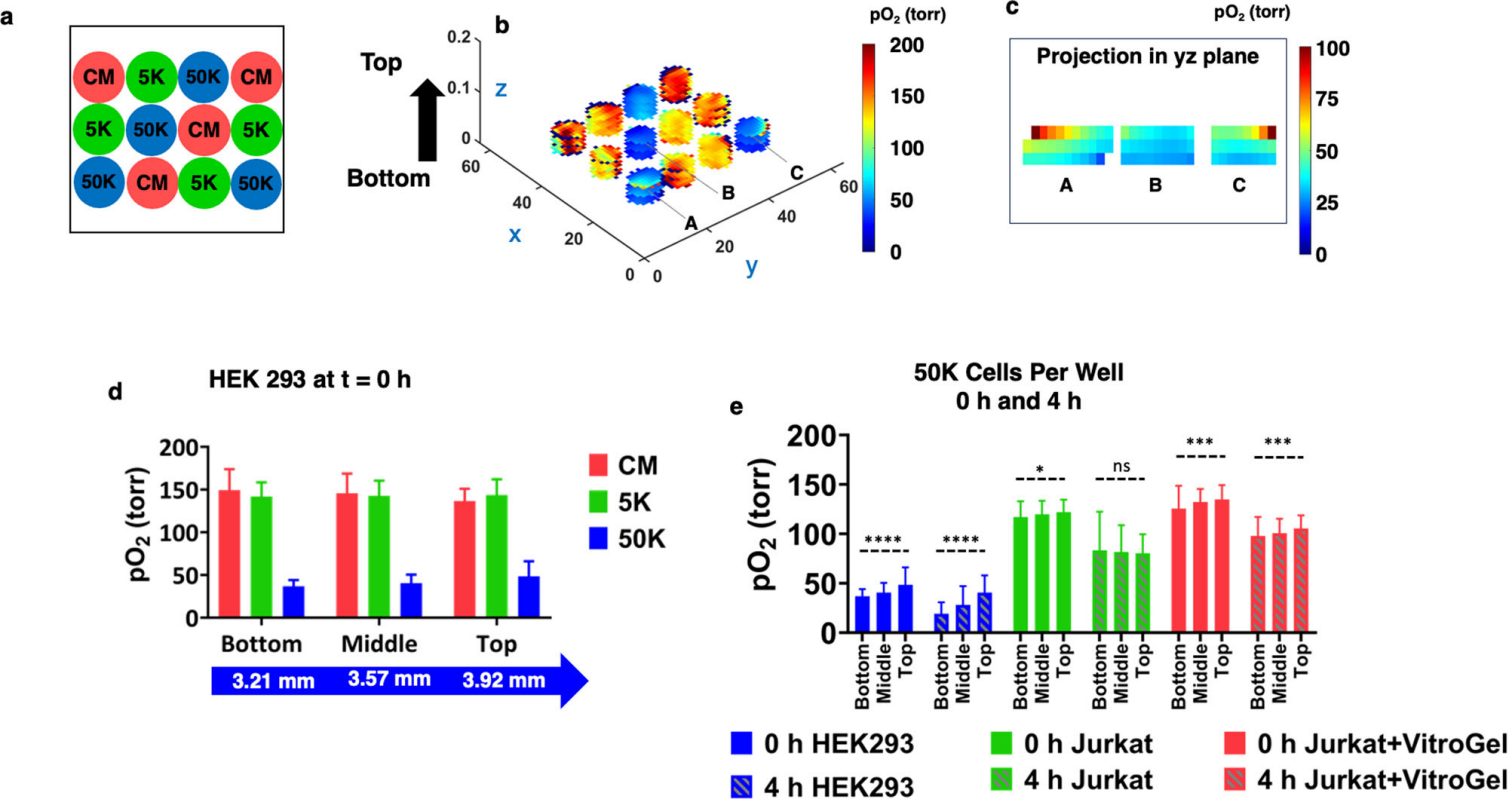
3D oxygen imaging of HEK-293 cells, Jurkat cells and Jurkat cells in VitroGel. **a** The cell seeding pattern of 0 K (CM), 5 K, and 50 K cells per well (*n* = 4). **b** Three slices from the bottom to top showing the change in pO_2_ over depth for HEK-293 cells. **c** pO_2_ maps of three wells with 50 K cells denoted as A, B, and C in **b** for better visualization of vertical gradient. For each well, the central slice in the yz plane was cut and reported. The central slice was identified by choosing the slice with the highest number of pixels in the “y” direction. **d** The pO_2_ at the bottom, middle, and top of the wells at t = 0 for CM, 5 K and 50 K HEK-293 cells. **e** The pO_2_ for 50 K cells at the bottom, middle and top of the wells for HEK-293, Jurkat and Jurkat seeded in VitroGel at t = 0 and 4 h. There is a significant difference in pO_2_ between the bottom of the well and the top of the well for all cases except for Jurkat cells at 4 h.

Finally, we demonstrate nondestructive 3D imaging of cells seeded in a scaffold using EPROI. Figure 9a shows the schematic of the three cell densities (0 K (CM), 5 K and 50 K cells per well). Figure 9b shows three slices of HEK-293 cells at 3.21 mm (Bottom), 3.57 mm (Middle), and 3.92 mm (Top) from the bottom. The three slices represent a complete 3D volume of the 150 μL of medium in each well. A gradient is visible from the bottom to the top of each well, especially for the wells with 50 K HEK-293 cells, which are adhered to the bottom of the well, as shown in Fig. 9c for three selected wells denoted as A, B, and C in Fig. 9b. Figure 9d shows the change in the pO_2_ values for control, 5 K and 50 K cells, going from the bottom of the well to the top. Figure 9e shows the change in pO_2_ at t = 0 and t = 4 h for HEK-293, Jurkat, and Jurkat in VitroGel for the 50 K cells. There is a statistically significant difference between pO_2_ at the bottom and top of the well (*p* ≤ 0.05) for all conditions except Jurkat at 4 h.

## Discussion

In this study, oxygen imaging of live cells was performed using trityl-OX071-based EPROI in an environment-controlled system, MWIR, that could accommodate 12 wells of a standard 96-well strip-well plate. The MWIR provided constant temperature, gas intake, and humidity in each well to maintain normal cell activity during the oxygen imaging of wells. While it is possible to perform point oxygen measurements using fluorescence-based probes, they are not suitable for 3D oxygen imaging. In addition, they are less sensitive at higher oxygen concentrations^24^. Once the MWIR system was established, oxygen imaging of HEK-293 cells was demonstrated using three cell densities: control (0 K), 5 K, and 50 K cells per well. We show that for metabolically active cells, the pO_2_ values of the wells are significantly different between different cell densities. After 24 h, the viability, pH and morphology of the cells that were in MWIR were similar to the cells that were in the BOD incubator, indicating that oxygen imaging did not interfere with the metabolic activity of the cells. A well-known limitation of MTT assay is that it doesn’t follow a linear relationship with the cell density at higher cell densities^55,56^. EPROI with the MWIR does not have this issue and could differentiate between different cell densities. To test the sensitivity of the method, oxygen imaging of HEK-293 cells was performed with six different cell densities (Fig. 5) and EPROI could differentiate between them. Longitudinal measurement of pO_2_ was also demonstrated. Experiments with non-adherent Jurkat cells showed higher pO_2_ values compared to HEK-293 adherent cells. The higher pO_2_ values may indicate a lower oxygen consumption profile for these cells.

EPROI is a 3D imaging method (Fig. 9) and was used to measure the difference in pO_2_ at different depths of cell culture wells and in 3D hydrogel scaffolds seeded with cells. The pO_2_ maps were heterogeneous in all cases, which could arise from the nonuniformity of oxygen consumption due to cell clustering within the wells.

We performed oxygen imaging with dead cells for comparison with live cells. We used two methods for cell killing, 4% PFA and 70 °C heat-shock. In both cases, the pO_2_ in wells with cells was higher than wells with live cells since dead cells do not consume oxygen. Interestingly, some cells were still alive after PFA treatment, and correspondingly these wells had lower pO_2_ compared to the medium, showing the sensitivity of the method. These results confirm that pO_2_ imaging is a sensitive tool that can differentiate between live and dead cells.

The oxygen imaging showed that cells seeded at high density experienced low pO_2_ in both the MWIR and BOD incubator. This finding has serious significance as, across the world, many labs may be subjecting their cells to low oxygen conditions without realizing it.

Finally, our experiments of Jurkat cells seeded in VitroGel (Fig. 8) demonstrated the ultimate promise of this technology, which is to assess the oxygen consumption and viability of cells seeded in a biomaterial without destroying the construct. Currently, there are no other methods to nondestructively assess oxygen consumption for cells in 3D for cells in tissue engineered constructs.

Oxygen consumption is one of the many ways to assess cell viability. Oxygen consumption can be determined by assessing the difference in oxygen supplied versus the amount expelled. Our method is based on a different principle. EPROI with MWIR measures the oxygen equilibrium in the medium where oxygen from the atmosphere is diffusing into the system from the top of the well while cells in the well consume oxygen, which causes a shift in the equilibrium to lower oxygen concentrations. This provides a snapshot of cell metabolic activity in real time without destroying the sample. This equilibrium provides a useful, noninvasive, 3D measure of cell viability and functionality that matters for cell therapy devices.

## Conclusions

In the current project, EPROI was demonstrated for cells in 96-well plates to be compatible with current cell culture practices. However, EPROI can be extended to other geometries by adjusting the instrument’s bore size, resonator size and design. The use of EPROI to assess cell viability and oxygen tension may lead to better devices for addressing unmet medical needs. This nondestructive oxygen imaging technique could be used to optimize therapeutic doses for cell therapies, optimize tissue engineered medical products, assess drug toxicity, and provide a new and innovative view of the live cells in these systems. EPROI is suitable for any tissue or organ of arbitrary sizes and may become a standard tool for quality control in cell therapy and tissue engineering.

## Materials and methods

We used two identical sets of 8-well strips from a 96-well strip well plate for all cell experiments. One set of three 8-well strips was used for oxygen imaging experiments using the MWIR system in JIAV-25™, while the second set of three 8-well strips was placed in the BOD incubator as a control. At the end of the experiments, an MTT assay was performed on the middle 12 wells in both sets to determine the cell viability in both sets. Student’s *T* test was used to calculate the statistical significance.

### Cells and culture conditions

Immortalized human embryonic kidney cell line HEK-293 (CRL-1573) and T-cell leukemia cell line Jurkat (TIB-152) were purchased from ATCC, Manassas, VA, USA. HEK-293 were cultured in high glucose DMEM medium (Gibco, ThermoFisher Scientific, Waltham, MA, USA) while Jurkat cells were cultured in the RPMI-1640 (Gibco, ThermoFisher Scientific, Waltham, MA, USA). Both medium were supplemented with fetal bovine serum (FBS, 10% by volume) and a mixture of penicillin 100 U/ml and streptomycin 100 μm/ml. Cells were seeded in 8-well strips in three densities: 0 cells (referred control medium, CM), 5000 cells/well (5K), and 50,000 cells/well (50K) in a volume of 150 µL of medium per well in a set seeding pattern (Fig. 3a) as well as other densities and patterns as demonstrated in the text. Wells without cells but with medium only (CM) served as an internal control. The dead cells were used as a negative control. No shaking culture was applied for experiments using Jurkat cells. Duplicate sets of 8-well strips with the same seeding densities and seeding pattern were kept in the BOD incubator as a control for the MWIR. The MWIR for oxygen concentration measurements and the BOD had a humidified atmosphere containing 5% (by volume) CO_2_ at 37 °C. No medium exchange was conducted during the measurements.

### Jurkat cells in VitroGel

Jurkat cell suspensions were cultured in 75-cm^2^ flasks in RPMI containing FBS with penicillin-streptomycin to a density of 1–2 × 10^6^ cells/mL. For experiments, cells were split and counted on a hemocytometer. Cells suspended in culture medium were mixed with VitroGel (glucose-based polysaccharide gel, TheWell Bioscience, Cat # VHM01) hydrogel matrix to achieve a final concentration of 5000 and 50,000 cells per 0.05 mL suspended in 20% by volume VitroGel diluted with cell culture medium. Cells in VitroGel were transferred to wells of a 96-well plate at 150 µL/well. After 15 min of incubation at room temperature inside the biosafety cabinet, 50 µl of additional complete RPMI medium was added. Cells were incubated overnight in the BOD incubator, and pO_2_ imaging was performed.

### Preparation of trityl stock solution

Trityl (Tris(8-carboxyl-2,2,6,6-tetra(2-(1-hydroxy-2,2-*d*_2_-ethyl))benzo[1,2-*d*:4,5-*d’*]bis(1,3)dithiol-4-yl)methyl **(**OX071**)**) radical (Fig. 1a) was used as an EPR oximetry probe for the measurement of oxygen concentration. One gram of trityl powder (MW: 1384.96, University of West Virginia) was dissolved in 8 ml deionized-double distilled water. This resultant suspension was vortexed gently, and pH was adjusted to 7.4 using 1.0 M NaOH. At pH 7.0, the trityl dissolved completely. Finally, the volume was made to 10 ml with deionized-double distilled water to reach to the 72.2 mM concentration and sterilized through a 0.22 μm syringe filter. Deoxygenation of the sterilized stock solution was done by bubbling 100% nitrogen gas via a needle through the rubber septum for 0.5 h to 3 h, after which the vial was sealed and stored at −20 °C. The stock was diluted to the desired concentration in sterile phosphate buffered saline (PBS, pH 7.4, Gibco, ThermoFisher Scientific, Waltham, MA, USA).

### Preparation of cell culture plate for pO_2_ imaging

Aliquots (1.5 uL) of sterile 100 mM OX071 stock were added to each well containing 150 uL of medium, which gives a final concentration of 1 mM OX071. Strips wells were then loaded onto the multi-well plate enclosure and sealed before placing them inside MWIR for imaging.

### pO_2_ calibration

The pO_2_ calibration of cell medium and VitroGel was performed in 1 mM OX071 by placing the sample in a 10 mm tube in JIVA-25^TM^ vertical 10 mm resonator at 37 °C. The sample was bubbled with nitrogen and air mixture at different gas concentrations to achieve the final desired equilibrium oxygen concentration between 0% and 21%. The inversion recovery sequence (Fig. 1b) and spectroscopic (no imaging) T_1_ provided average pO_2_ values and were used to obtain pO_2_ versus R_1_ calibration (*26, 29*). The pO_2_ calibration details (Fig. 1e) are as follows: O_2_ relaxation rate in PBS at 0 mmHg was 0.181 Ms^−1^, with a slope of 109.664 torr/Ms^−1^, in DMEM cell medium at 0 mmHg was 0.1537 Ms^−1^, with a slope of 123.3941 torr/Ms^−1^. The O_2_ relaxation rate in VitroGel made with DMEM cell medium at 0 mmHg was 0.130 Ms-1, with a slope of 113.484 torr/Ms^−1^.

### pO_2_ measurement and image processing

Oxygen imaging experiments were performed using JIVA-25™ at the resonance frequency of 720 MHz. The conditions in the MWIR were a humidified gas mixture of 95% air and 5% CO_2_ at 37 °C throughout the pO_2_ measurements. To avoid contamination, the incoming gases were filtered using inline filters, and autoclaved deionized water was used in the humidifier. Any hardware that came into contact with the strip wells, such as the multi-well plate cover was cleaned with 70% (by volume) ethanol. pO_2_ images were acquired using the pulse inversion recovery electron spin echo (IRESE) sequence^27,30^. Prior to acquiring pO_2_ maps, an amplitude map image was acquired using an electron spin echo (ESE) sequence that shows the signal amplitude for each well. The imaging parameters were: 90°/180° pulse lengths 60 ns, 16-phase cycles scheme with free induction decay (FID) suppression, spin-echo delay 400 ns, equal solid angle spaced 654 projections, 67 baselines, 1.5 G/cm gradient, 10-time delays from 410 ns to 15 μs (410 ns, 612 ns, 912 ns, 1.361 μs, 2.03 μs, 3.029 μs, 4.518 μs, 6.74 μs, 10.055 μs, 15 μs), 55 μs repetition time, overall, 10 min image duration. R_1_ (1/T_1_) images were reconstructed using filtered back projection in an isotropic 64 × 64 x 64 cube, field of view 5 cm × 5 cm x 5 cm with 0.78 mm cubic voxels. Approx. 6–7 slices from the bottom to the top of the well and about ~ 300–800 voxels per slice were collected from each well. The R_1_ maps were calculated voxel by voxel by fitting data to the single exponential fit and converted to pO_2_ maps using the calibration values obtained from the calibration experiment.

### Data collection

The wells of 96-well plates are cylindrical with a 12.15 mm height and 6.25 mm diameter (Fig. 9B, C). In a well, 150 µL of aqueous medium has a 5.98 mm height. A 50 mm cube of pO_2_ data containing 262,144 voxels was collected that encompassed the 12 wells of the 96-well strip wells. To create pO_2_ images for figures, a mask was created using a spatial image that fits within the bounds of the well. This mask was then transferred to pO_2_ maps to obtain the images and image statistics using ArbuzGUI developed by Dr. Boris Epel at the University of Chicago^57^. The typical number of voxels ranged from ~300 to ~800 per well. The box plot pO_2_ represents the mean, median, and standard error from all slices of the wells representing each cell density.

### Microscopy

Brightfield microscopy (Leica DMi1, Leica Microsystems) was used to confirm the cell morphology before and after oxygen imaging.

### pH measurements

The pH was measured for cell culture medium 1) taken from cells immediately following EPROI imaging in JIVA-25 or 2) taken from cells that were incubated in the BOD incubator (control). Medium from the cell culture wells (96-well stripwell plates) were pooled into the 1.5 mL vials for measuring pH (Jenway 3501 pH meter).

### MTT cell viability assay

To validate cell viability at the end of oxygen imaging, MTT assay (Abcam, Waltham, MA, USA, Cat # ab211091) was performed on cells that were imaged by EPROI and compared with the control cells incubated in a BOD incubator for the same duration. Briefly, 1 mM trityl (OX071) was added to the HEK-293 cells plated in different densities in 8-well strips. At the end of treatment, medium containing trityl was removed, and cells were gently washed with PBS. MTT solution (50 µL MTT Reagent + 50 µL cell culture medium) was added and incubated for 3 h at 37 °C. The plate was wrapped in aluminum foil and shaken on an orbital shaker for 15 min at 350 rpm. Thereafter, the absorbance was measured at 590 nm in a microtiter plate reader (BioTek Synergy LX multimode reader).

### Trypan blue assay for dead cell experiment

5 K and 50 K HEK-293 cells were seeded in a 96-well plate. 24 h later, cells were killed either by incubating in the presence of 4% paraformaldehyde (PFA) for 30 min at room temperature or by incubating at 70 °C for 15 min. Trypan Blue exclusion assay was done to confirm cell death. Spent medium was aspirated and cells were gently washed with 150 µL PBS. Trypan blue (0.4%, 50 µL) (ThermoFisher Scientific, Cat number 15250061) was added to the well and incubated for 3 min at room temperature. Excess trypan blue was removed, and cells were visualized under the microscope (Leica DMi1 Inverted Phase Contrast Microscope). Viable cells remained unstained, whereas dead cells appeared blue.

### Statistical analyses

pO_2_ data are presented as mean with a standard error or as box plots showing four quartiles. MTT data are presented as mean with standard deviation. Student’s t-test was performed and expressed as *p ≤ 0.05, **p ≤ 0.01, ***p ≤ 0.001, ****p ≤ 0.0001, ns: *p* > 0.05 not significant.

## Data Availability

The datasets used and analyzed during the current study are available from the corresponding author upon reasonable request.
