# Author Correction: Nondestructive, longitudinal, 3D oxygen imaging of cells in a multi-well plate using pulse electron paramagnetic resonance imaging

**DOI:** 10.1038/s44303-024-00016-4

**Published:** 2024-04-10

**Authors:** Safa Hameed, Navin Viswakarma, Greta Babakhanova, Carl G. Simon, Boris Epel, Mrignayani Kotecha

**Affiliations:** 1 Oxygen Measurement Core, O2M Technologies, LLC, 2201 W Campbell Park Dr, Chicago, IL 60612 USA; 2 Biosystems and Biomaterials Division, National Institute of Standards and Technology, 100 Bureau Drive, Gaithersburg, MD 20899 USA; 3 Department of Radiation and Cellular Oncology, The University of Chicago, 5841 S Maryland Ave, Chicago, IL 60637 USA

**Keywords:** Biotechnology, Biomaterials, Cellular imaging, Magnetic resonance imaging

Correction to: *npj Imaging* 10.1038/s44303-024-00013-7, published online 01 April 2024

The abstract for this article was shortened to fit the Author list and affiliation. The original article has been corrected.

